# Enhancement of Dynamic Damping in Eco-Friendly Railway Concrete Sleepers Using Waste-Tyre Crumb Rubber

**DOI:** 10.3390/ma11071169

**Published:** 2018-07-09

**Authors:** Sakdirat Kaewunruen, Dan Li, Yu Chen, Zhechun Xiang

**Affiliations:** 1 Birmingham Centre for Railway Research and Education, University of Birmingham, Birmingham B152TT, UK; 2 Department of Civil Engineering, School of Engineering, University of Birmingham, Birmingham B152TT, UK; DXL561@student.bham.ac.uk (D.L.); chenyu304304@outlook.com (Y.C.); 15372291781@163.com (Z.X.)

**Keywords:** crumb rubber, high-strength concrete, damping, dynamic moduli, railway application, recycled material

## Abstract

There is no doubt that the use of waste rubber in concrete applications is a genius alternative because Styrene is the main component of rubber, which has a strong toxicity and is harmful to humans. Therefore, it will significantly reduce impacts on the environment when waste rubber can be recycled for genuine uses. In this paper, the dynamic properties of high-strength rubberised concrete have been investigated by carrying out various experiments to retain the compressive strength, tensile strength, flexural strength, electrical resistivity, and damping characteristics by replacing fine aggregates with micro-scale crumb rubber. Over 20 variations of concrete mixes have been performed. The experimental results confirm that a decrease in the compressive strength can be expected when the rubber content is increased. The new findings demonstrate that the high-strength concrete can be enhanced by optimal rubber particles in order to improve splitting tensile and flexural strengths, damping properties, and electrical resistivity. It is therefore recommended to consider the use of rubberised concrete (up to 10 wt. % crumb rubber) in designing railway sleepers as this will improve the service life of railway track systems and reduce wastes to the environment.

## 1. Introduction

At the beginning of the last century, with the development of science and technology, the reduction of forest resources led to the production of steel and concrete railway sleepers rather than wood [1,2,3]. Since railway sleepers are effective but expensive, reinforced concrete has been used as a reliable and more efficient material for railway sleepers since the 1950s [4]. On the other hand, the rapid development of the automotive industry has led to the rapid development of the rubber tire industry and brought about the dilemma of how to deal with rubber waste that poses a serious threat to the environment.

Recently, many researches have proposed and encouraged the use of waste rubber in concrete to improve its performance [5,6,7]. More precisely, in the railway sector the rubber concrete sleepers have known a high-demand due to its efficiency in high-intensity pressure vibration environment. In this study, the crumb rubber is replacing the fine aggregates in the concrete sleepers in order to evaluate their ability to absorb the vibration energy. While it is expected to have a material with better performances than conventional concrete, the reason behind this evaluation is to create a rubber concrete that can maintain its strength at an acceptable level, thus enhancing the performance and sustainability of the railway track systems exposed to high-intensity impact loading conditions [8,9,10].

The purpose of this paper is to highlight the sensitivity of dynamic properties of the high-strength concrete to crumb rubber inclusion as well as its capability in meeting the standards of railway concrete sleepers’ manufacturing. Different proportions of crumb rubber with different particle sizes (75, 180, and 400 µm) have been used in order to analyse their effects on the compressive strength, splitting tensile and flexural strengths, electrical resistivity and damping properties of the rubberised concrete.

## 2. Railway Applications

**Figure 1:**
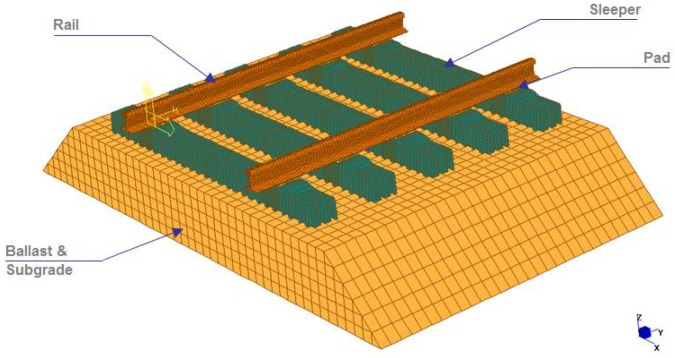
Components of railway tracks.

Conventional rail track structure can be divided into superstructure and substructure (Figure 1). The superstructure includes rails, rail pads, fastening system and rail sleepers. The substructure consists of ballast, sub-ballast and formation. Railway sleeper is one of the most important safety-critical components of the track structure. The main functions of rail sleeper are to transfer vertical loads from rails to foundation and maintain rail gauge. According to Cai [11], the sleepers are laid horizontally under the rail and placed on the track bed, bearing the pressure from the rail and spread it all over the ballast bed.

The sleepers are usually manufactured by timber or concrete composite nowadays. Timber sleepers are often used in North America which have superior elasticity, better mitigation and absorption of vibration, but their life cycle is no more able to meet today’s market requirements. Consequently, concrete sleepers have recently become the most widely used due to their low maintenance cost and longer life cycle in comparison with timber sleepers [12].

The railway concrete sleepers are expected to service for 50 years [13,14]. However, in practice the service life of concrete sleepers barely meet the design requirement due to many causes. In order to determine the total energy needed for the failure of railway concrete sleepers under sudden load, their dynamic response was investigated. It has been found that the railway concrete sleepers’ impact resistance was more affected under splitting mode as there was a lack of bonding between concrete and steel bars under dynamic circumstances [15]. The most critical problems related to failure of concrete sleepers were surveyed by Stuart. The survey reported that concrete sleepers cracking from dynamic loads were significant [16]. Their durability is reduced as the cracks start to appear which increase the chance of fatigue failure. The cracks in sleepers are initiated when they experience severe loading conditions such as high-magnitude wheel loads created by ruthless wheels or rail abnormalities [17]. It has therefore been observed that the use of crumb rubber in concrete could minimize the damage of cracks in sleepers, as the crumb rubber concrete has shown significant toughness properties and impact resistance. The elastic properties and fatigue resistance will also improve by using crumb rubber in concrete [18]. The previous research conducted by Sallam et al. [19] revealed that resistance of crack under impact loading will be improved in the presence of rubber particle. Hameed and Shashikala [18] indicated that the stiffness of concrete will be increased when containing crumb rubber under cyclic loads. The outcome of this research has indicated the dynamic property of crumb rubber concrete through various experiments which provide important data for future railway sleeper durability design.

## 3. Methods

### 3.1. Rubber Wastes and Their Applications

**Figure 2:**
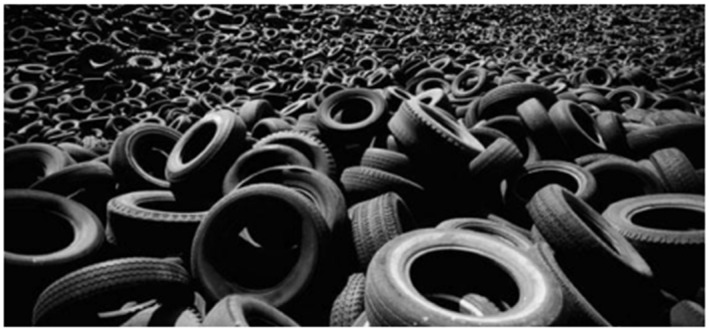
Waste tyres.

The large amount of abandoned waste tyres (Figure 2) while handled and removed quickly will certainly impact the environment. Common solid waste disposal methods like incinerated and landfilled are not suitable for this kind of waste as they lead to the release of toxic substances which results in serious contaminations of underground water systems. The thousands of hectares of landfills not only occupy sites that can have better usages, but also contribute to bacteria and mosquitoes breeding, causing infectious diseases and blazes [20].

**Figure 3:**
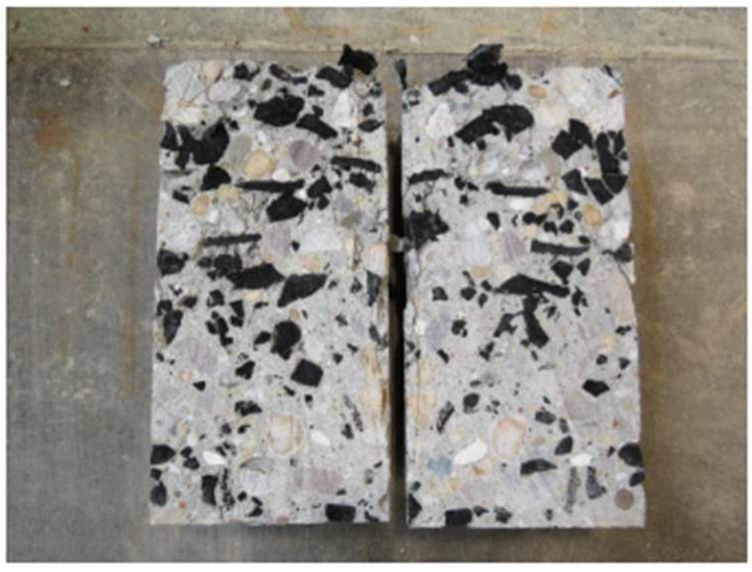
Crumb rubber concrete.

As early as 1976, there was a patent for old tyres usage in the United States [21,22]. Nowadays, more granulation methods appeared, such as extraction of steel fibres from waste tyres as reinforcement in concrete is now possible [6]. Some researchers reported that the crumb rubber, when added to ballasted tracks, resulted in better absorption of the vibration impact energy and improved their life cycle [23]. More researches devoted to the possibility of partially replacing aggregates with rubber particles (Figure 3) as an eco-friendly alternative while dealing with rubber waste [24], and also improving the concrete damping characteristics [25].

### 3.2. Relevant Previous Studies

Most studies have shown that the replacement of the fine aggregates by crumb rubber in concrete will reduce its strength to a certain extent, and the degree of decrease in strength depends on both the particle size and the amount of addition [26]. Still, other studies reported many other benefits of using rubber in concrete. Rubber particles can act as an air-entraining agent and hence providing concrete with a better freeze-cycle protection [27]. Thermal resistance also presents a challenge for concrete sleepers, and since rubber has good heat insulation it was found that the crumb rubber can increase the thermal resistance of concrete to about 18.52% [28]. Conventional concrete has better fire resistance than crumb rubber concrete as the rubber is a flammable material and its particles on the concrete surfaces can easily be burnt [29]. The dynamic properties are very important in the railway track design process. For concrete sleepers, cracking could be caused by wheel–rail interaction force which can be divided into dynamic load and impact load [30]. Dynamic load is wheel–rail interaction under normal situation, whereas impact load is also wheel–rail interaction caused by defects in wheel or rail. In durability design, sleepers need to withstand dynamic load and impact load. However, the dynamic testing data of crumb rubber concrete is not enough for concrete sleeper design. Therefore, investigation of dynamic properties is necessary for new material concrete sleeper design [31].

## 4. Materials

The emphasis of this study is placed on the application of recycled crumb rubber to enhance dynamic damping of structural concrete for railway applications. To enable the industry application, specific requirements must be complied (e.g., EN 13230 [32]). The materials for the concrete mix are thus specifically chosen to meet standard requirements.

### 4.1. Cement

As described in BS EN 197-1 [33], the Portland Cement Type I (CEM I) could be used to mix concrete which has a characteristic strength of 55 MPa.

### 4.2. Silica Fume

Silica fume is a grey powder that belongs to a broad class of siliceous and aluminous materials called “Pozzolans”, which plays a particular role in filling the pores between the cement. Adding the right amount of silica fume contributes to improving the compressive and flexural strengths, impermeability, corrosion/impact resistances. These improvements are due to the consummation of calcium hydroxides, produced by the hydration of cement, by the silicon dioxide SiO_2_ [34] present in the composition of the silica fume as summarized in Table 1. The use of silica fume in this study is to compensate the loss of strength when crumb rubber is used.

### 4.3. Coarse Aggregates

Coarse aggregate refers to pebbles and gravel, while fine aggregate refers to natural sand and artificial sand. Aggregates with a particle size greater than 4.75 mm are called coarse aggregates and aggregates with a diameter of 4.75 mm or less are called fine aggregates. Using a vibrating sieve to screen out usable gravels, the gradation table is produced as shown in Table 2. For concrete sleepers, all aggregates should comply with BS EN 12620 [36].

### 4.4. Water

The water used to mix concrete should be clean and easy to obtain on site, in accordance to BS EN 206-1 [37].

### 4.5. Admixture

Concrete admixtures are substances, once added to the mixture, that improve and adjust the performance of concrete. Typically, according to BS EN 934-2 [33], they cannot be added in excess of 5% of the cement content. The most common such admixtures are water-reducing agents, set retarder, air-entraining admixture, etc.

### 4.6. Concrete

The general performance of concrete sleepers should conform to BS EN 206-1 [37], which means cylinder samples compressive strength not below 55 MPa, cement content over 300 kg/m^3^ and a maximum water–cement ratio of 0.45 cannot be exceeded. After casting, the concrete samples must be covered with plastic sheet for 24 h, and then placed in a water tank for 7 and 28 days prior for testing. The mix design (see Table 3) has been further developed from previous studies to ensure that the concrete could satisfy the minimum requirements [32,33,38]. A pilot test has been conducted to achieve the requirement and reveal an appropriate ratio between water and binder (cement + silica fume). The ration has been retained for all mixes to ensure that the comparison could be rationalised.

## 5. Experimental Results and Discussion

**Figure 4:**
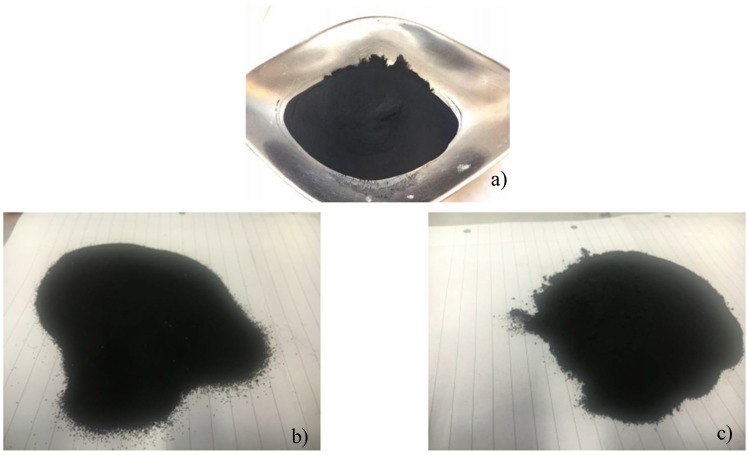
Rubber Powder ((**a**) 75 µm; (**b**) 400 µm; and (**c**) 180 µm particle size, respectively).

Based on the design requirements of the railway concrete sleepers that comply with the British Standards BS EN 206-1 [37], and on the theory described in the “Design of Normal Concrete Mixes” [39], 133 specimens were casted using different materials’ proportions. Silica fume is used as a partial replacement of the cement content and the crumb rubber as an alternative aggregate by replacing different amounts of fine aggregates. One hundred and fourteen specimens were characterized by the use of the crumb rubber, half of them were made using a mix of 180 and 400 micron crumb rubber (Mixes A), and the other half was mixed using 75 micron crumb rubber (Mixes B), as shown in Figure 4 and Table 3.

**Figure 5:**
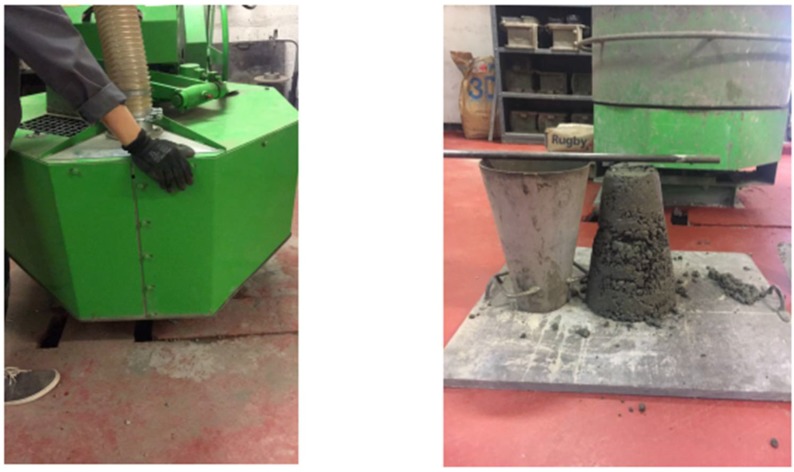
Concrete mixer (**left**); concrete slump test (**right**).

**Figure 6:**
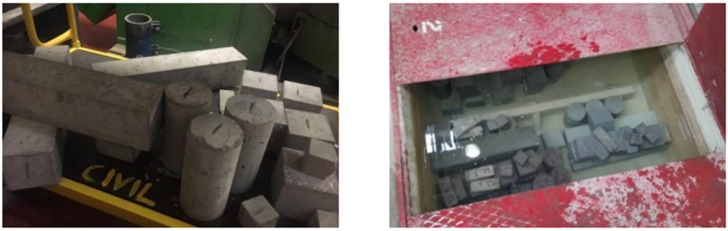
Specimen shapes (**left**); curing tank (**right**).

Serviceability tests were conducted after each mix as shown in Figure 5. Then, they were cured in water (see Figure 6). For each single batch, there were 19 samples manufactured. Once the concrete has been mixed, it was poured directly into different shaped moulds: 100 mm cube, Φ100 × L200 mm cylinder, W100 × H100 × L500 mm prism, and W45 × H20 × L120 mm prism, each shape corresponding to the compressive strength testing (CS), splitting tensile strength testing (STS), flexural strength testing (FS), and electrical resistivity testing (ER) and vibration testing (VT), respectively as shown in Table 4. In order to provide good curing conditions, the samples are demoulded after 24 h and placed in a tank at appropriate temperature, then removed after 7 and 28 days respectively to carry out tests.

### 5.1. Compressive Strength

**Figure 7:**
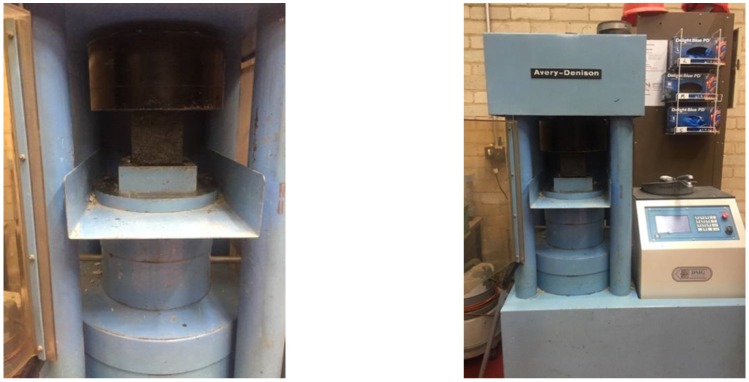
Compressive test setup.

**Figure 8:**
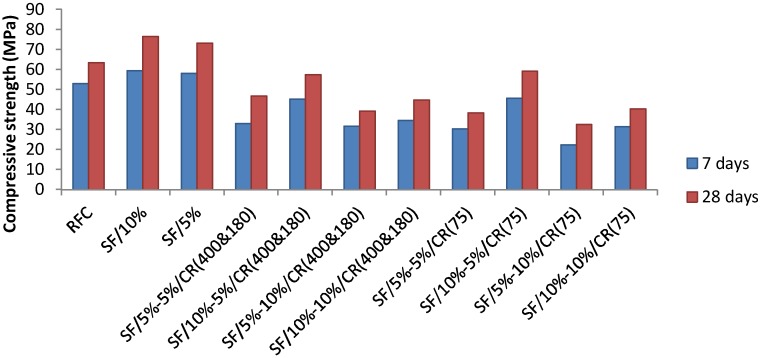
Compressive strength of crumb rubber concrete.

The compressive strength tests (Figure 7) were carried out according to BS EN 12390-3 [40]. With a total of six cube samples per batch, previously removed from the tank and placed in an oven one day in advance in order to dry, three samples were tested after 7 days and the other half after 28 days. The results of the compressive tests are summarised in Figure 8.

According to Figure 8, the results of compressive strength at 7 days and 28 days of experimental samples is shown. The reference concrete showed a compressive strength of 53.05 MPa at 7 days and 63.33 MPa at 28 days which shows good performance in compressive strength. The highest performance of compressive strength are the samples containing 5% and 10% silica fume, with 73.13 MPa and 76.5 MPa, respectively. Many previous literatures have indicated that the compressive strength would reduce in replacement of fine aggregate with crumb rubber. The results of this research also confirmed this phenomenon. In fact, all the samples with crumb rubber have less performance in compressive strength. There are only 2 samples containing crumb rubber that met the design requirements (55 MPa) according to BS EN 206-1 [37] in which they are 10% silica fume/5% 75 micro-crumb rubber and 10% silica fume/5% 400&180 micro-crumb rubber. The best performance of compressive strength crumb rubber concrete sample is 10% silica fume/5% 75 micro-crumb rubber which has 59.2 MPa compressive strength at 28 days and it is only 6.5% lower than reference concrete.

The 10% silica fume or 5% 400&180 micro-crumb rubber has a similar result which is 57.3 MPa. Obviously, the addition of crumb rubber negatively affected the concrete performance while the silica fume improved it. This has been proved by different authors who establish a proportional relation between the increase of crumb rubber content in the concrete mixture and the reduction in its compressive strength [41,42,43]. However, the rubber concrete containing a proportion of 10% silica fume and 5% crumb rubber has shown considerable results by reaching 59.20 MPa and 57.30 MPa at 28 days in both cases: 75 micron and the mix of 180 and 400 micron, respectively. The fact that the addition of 75 micron crumb rubber results in higher concrete compressive strength comparing to the mix of 180 and 400 micron by approximately 3.3% is due to the finer rubber particles that have a better ability in filling voids, thus offering a higher compressive strength [44]. Therefore, it has been deducted from the tests’ results of this study that the crumb rubber concrete can be considered as a reliable material for railway concrete sleepers, since its compressive strength meet the standard for sleepers manufacturing (55 MPa).

### 5.2. Splitting Tensile and Flexural Strengths

**Figure 9:**
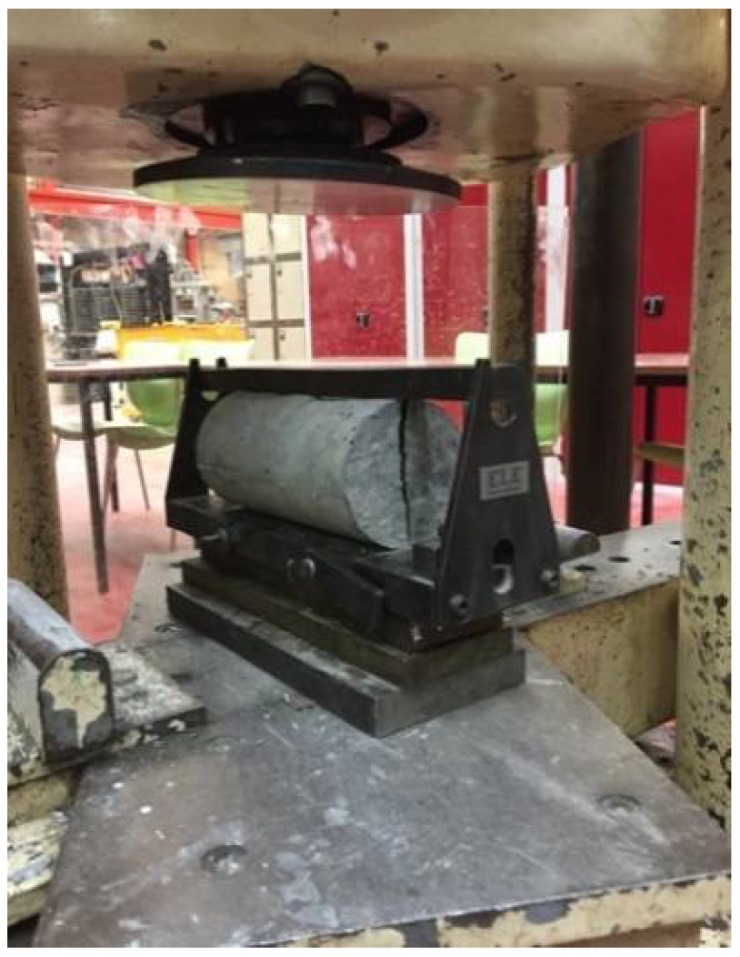
Splitting tensile test.

According to BS EN 12390-6 [45], cylindrical specimens are used to test the rubber concrete splitting test after 28 days. In order to stabilize the specimens during the test, a pad is placed between the bearing surface and the press plate of the machine forming a corresponding strip loading between the top and the bottom of the specimens (Figure 9). At the beginning of the test, the loading was carried out continuously and uniformly at a speed ranging from 0.02 to 0.05 MPa/s until it reached 30 N/mm^2^, the loading speed was then increased to range between 0.05 and 0.08 MPa/s until the end of the test. The test is stopped when the specimen is damaged and the damage load is recorded at an accuracy of 0.01 KN.

**Figure 10:**
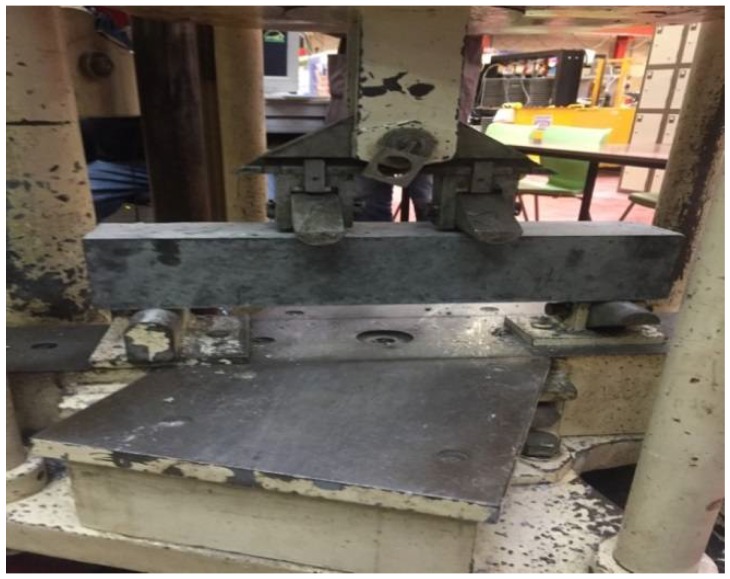
4-point bending test.

**Figure 11:**
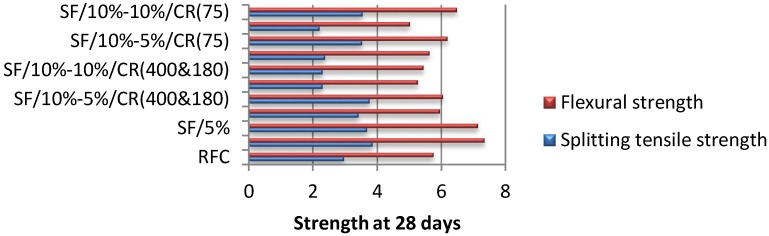
Splitting tensile strength of crumb rubber concrete.

On the other hand, in order to measure the flexural strength of the rubberised concrete, several 4-point bending tests (Figure 10) have been carried out on prism specimens at 28 days according to BS EN 12390-5 [46]. The specimens are placed on two roller supports and loaded from an upper roller at a constant rate of 0.05 MPa/s, and the tests’ results stop recording once the specimens fail. The results of both tests are presented in each graph of Figure 11.

According to BS EN 12390-6 [45], the splitting tensile strength of concrete at 28 days was calculated using the function below:

(1) $$f_{ct}=\frac{2F}{\pi LD}$$

where *f_ct_* is the splitting tensile strength (MPa), *&* is the maximum load (N), *L* is the length of the line of contact of the specimen (mm), and *D* is the designated cross-sectional dimension (mm).

Similarly to the compressive strength results, the increase in silica fume content clearly improved the splitting tensile strength by 30% comparing to conventional concrete, reaching a maximum value of 3.85 MPa at 28 days as shown in Figure 11. Also, as expected from previous studies, the rubber powder replacing fine aggregates has considerably decreased the splitting tensile strength, especially when its amount is increased to 10% and mixed with 5% silica fume, leading to a minimum value lower than the one obtained from the conventional concrete by 35% in the case of 75 micro rubber, and 30% in the case of mixing 180 and 400 micro rubber. Though, the proportions that seemed to show better performance than normal concrete are the ones containing 10% silica fume: mixed with 5% crumb rubber in the case of 180 and 400 micro rubber, and 10% in the case of 75 micro rubber (3.75 MPa and 3.54 MPa respectively).

Likewise, the flexural strength has been calculated following the British standard BS EN 12390-5 [46]:

(2) $$f_{cf}=\frac{FI}{d_{1}d_{2}^{2}}$$

where *f_cf_* is the flexural strength (MPa), *F* is the maximum load (N), *I* is the length between roller supports (mm), and *d*_1_ and *d*_2_ are the cross-sectional dimensions of specimen (mm).

As shown in Figure 11, similar trend was observed between the splitting tensile and the flexural strengths. The maximum value recorded is 7.34 MPa and the minimum is 5.02 MPa, both correspond to SF10%/CR0% and SF5%/CR10%, respectively. Same observations have been deducted in the flexural test, which are the improvement of concrete strength by increasing the silica fume content and decreasing the crumb rubber proportion, and its deterioration in the opposite way. Many previous studies have come to similar conclusions regarding both, splitting tensile and flexural strengths of rubberised concrete, when replacing normal coarse aggregates with recycled rubber crumb [21,47,48]. Yet, the specimens containing a combination of 10% silica fume and whether 5% of the 180&400 micro rubber or 10% of 75 micro rubber have shown acceptable flexural strengths superior to plain concrete by 8% and 13%: 6.19 MPa and 6.49 MPa, respectively.

The tensile and flexural ratio can be calculated by:

(3) $$tensile\;ratio=\frac{f_{t}^{\prime }}{f_{c}^{\prime }}*100\%$$

(4) $$tensile\;ratio=\frac{f_{f}^{\prime }}{f_{c}^{\prime }}*100\%$$

**Figure 12:**
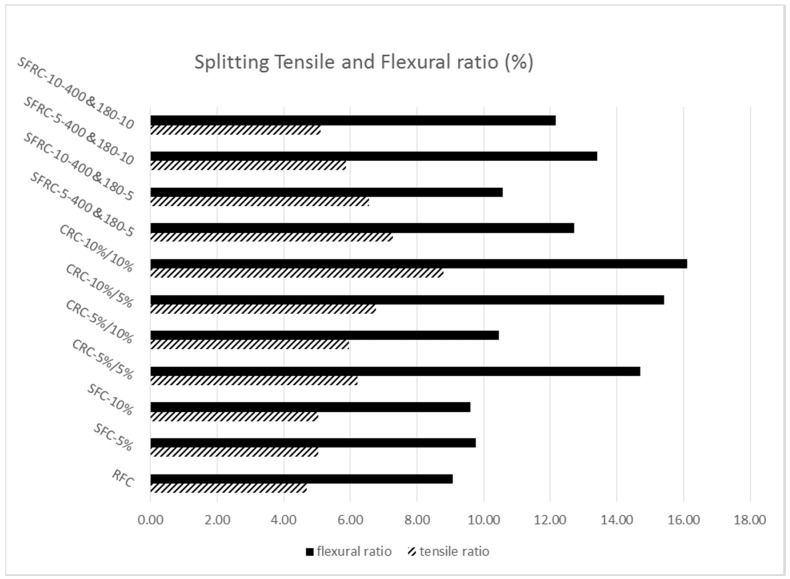
Splitting tensile and flexural ratio of crumb rubber concrete.

The tensile ratio and flexural ratio have been calculated by Formulas (3) and (4) shown in Figure 12. Both the tensile and flexural ratio follow the same trend as flexural/splitting tensile strength. From the graph, all the flexural ratio of crumb rubber concrete are more than 10%. The highest tensile/flexural ratio is 10% silica fume/10% 75 micro rubber, which are 8.78% and 16.10%, respectively. Non-crumb rubber samples show very low tensile ratio, they are: 4.69% for reference concrete, 5.03% for 5% silica fume concrete and 5.03% for 10% silica fume; 10% silica fume/10% 400 & 180 micro-crumb rubber has similar tensile ratio with non-crumb rubber sample, which is 5.11%.

### 5.3. Electrical Resistivity

**Figure 13:**
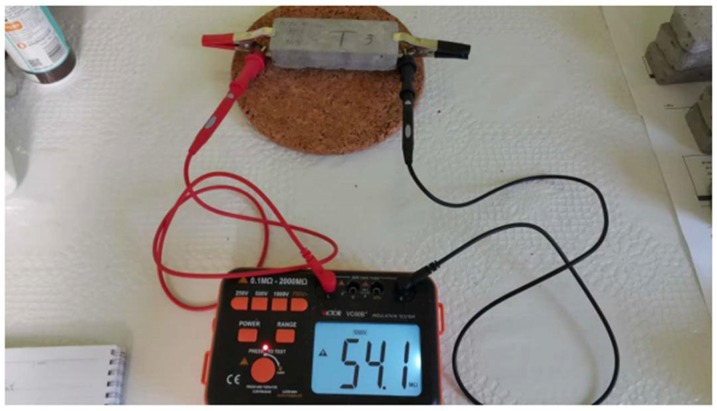
Electrical resistivity test.

Generally, there are two main methods to measure the electrical resistance of cementitious materials: two-electrode method and four-electrode method [49]. In this study, the two-electrode method has been considered to test prism specimens at 28 days, after having been removed from the tank for natural drying prior for testing. Victor VC60B+, an insulation tester, has been used to determine the electrical resistance (ohm) of the specimens, by connecting leads to both their ends and setting the voltage at a constant value of 1000 Volts as shown in Figure 13. The results of the experiments, shown in Figure 12, are recorded 60 s later.

After 28 days, the electrical resistance was measured then calculated using the resistance equation [22]:

(5) $$\rho =k*R;k=\frac{A}{L}$$

where *R* is the resistance of concrete, *k* is a geometrical factor which depends on the size and shape of the specimen, *A* is the cross-sectional area perpendicular to the current, *L* is the height of the specimen.

**Figure 14:**
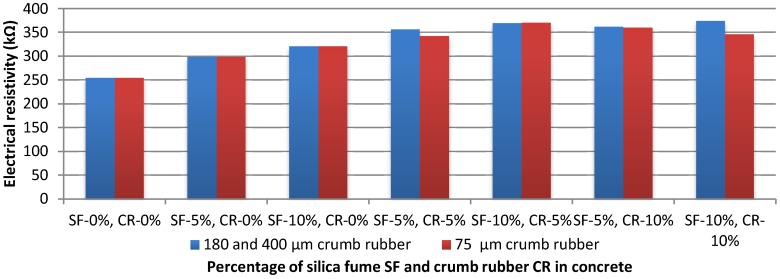
Electrical resistivity of rubber concrete with different particles size.

As it can be seen from Figure 14, the plain concrete specimen has the lowest electrical resistivity among all others: 255 kΩ. The concrete’s electrical resistivity increased once the silica fume has been added, which proved what has been found by other researchers [23,50,51]. Obviously, as the rubber is considered as a good insulator, the concrete samples containing crumb rubber have shown the best results in terms of electrical resistivity: 374.25 kΩ and 371 kΩ for SF10%/CR10% (180 & 400) and SF10%/CR5% (75) respectively. Therefore, the addition of crumb rubber is capable of enhancing the electrical resistivity of plain concrete by 47%, and by 17% if it contained 10% of silica fume. In fact, both insulator materials (silica fume and rubber) have shown good performance as expected, due to their ability to prevent the two electrodes to transmit current in concrete [52].

### 5.4. Vibration Damping

**Figure 15:**
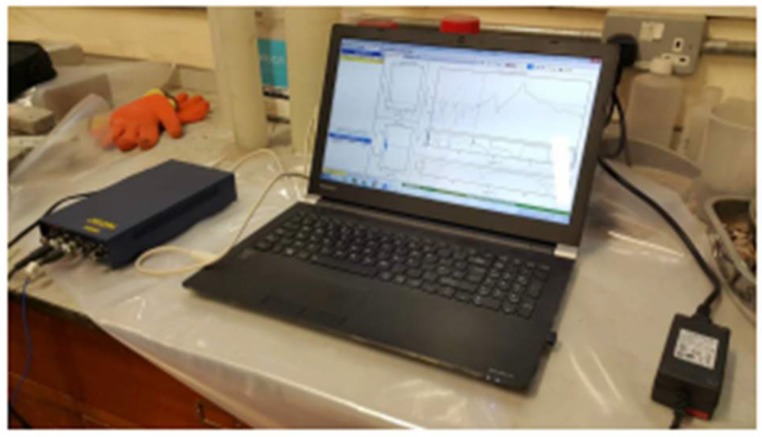
DATS modal analysis suite.

**Figure 16:**
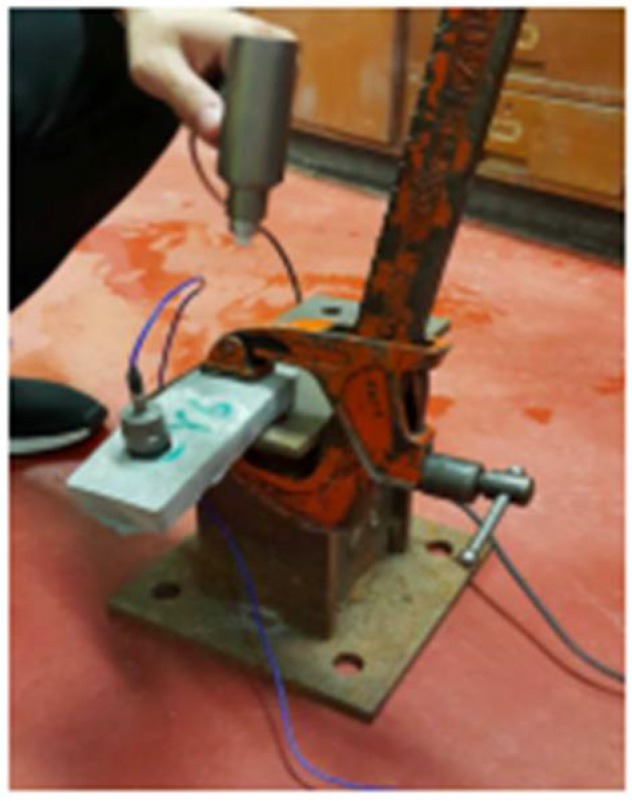
Damping test.

**Figure 17:**
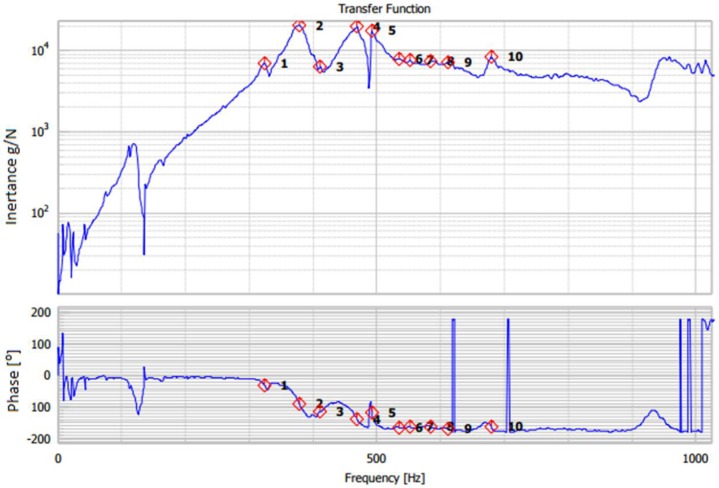
Frequency response function.

The vibration tests have been conducted in the civil engineering laboratory to avoid vibration from surrounding environment. Prism specimens were used to carry out these experiments using the following equipment: computer with PROSIG hammer system, DATS modal analysis software, and an accelerometer. The specimens’ surfaces have been cleaned to eliminate bias in tests results [52,53], and a stable support (Figure 15) was used to fix the samples as a cantilever beam since placing the stationary platform on the concrete floor makes the resonant frequency relatively high. The vibration of samples was excited by a PCB hammer (Figure 16) at a frequency range of 0 to 1600 Hz [54,55], and as shown in Figure 16, a 5-order average vibration response of each sample is presented by the frequency response function (FRF). The graph of time series is illustrated in Figure 17.

Generally, there are two methods to analyse the damping ratio and both have been considered in this study. The first method is called exponential curve fitting method (ECFM); this method is based on calculating the damping ratio directly using the natural frequency and the vibration response of the concrete samples from the FRF graph, as can be seen from Figure 17. The specimen containing 10% silica fume has a natural frequency of 469 Hz, and the one containing the same proportion of silica fume in addition to 5% of crumb rubber (75 micron) has 500 Hz of natural frequency. All the natural frequencies recorded during the tests are presented in Table 5, although, factors such as the high stiffness of concrete and thickness of the metal gasket can affect the range of the natural frequencies [52,56].

**Figure 18:**
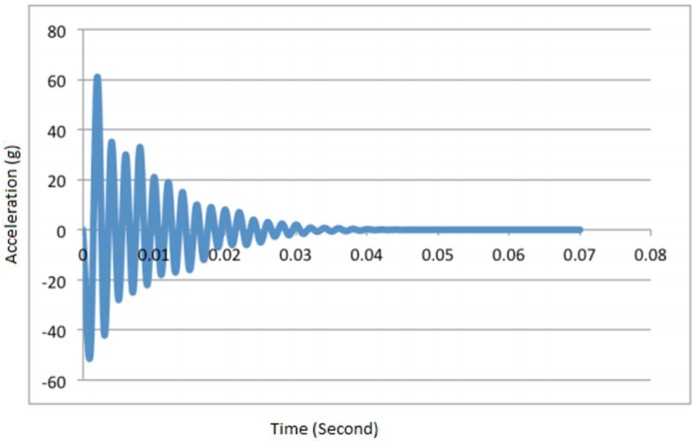
Vibration response of the concrete.

The acceleration-time graph is then plotted and the exponential line is fitted with the peak amplitude according to the decreasing trend of the vibration response, while the equation of the exponential line is generated from the Microsoft Excel program as illustrated in Figure 18.

**Figure 19:**
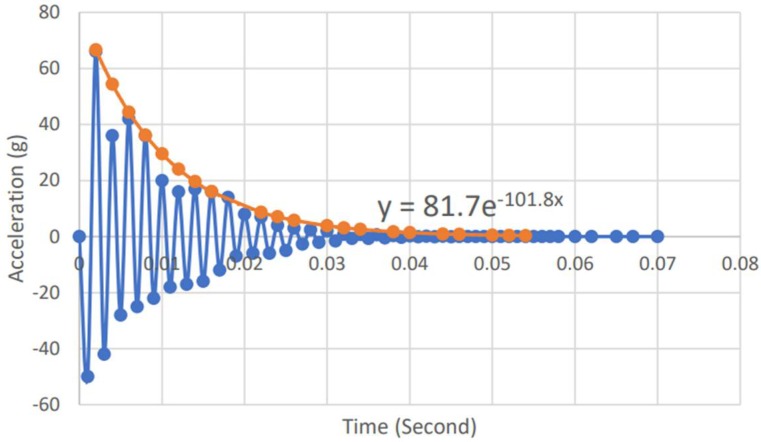
Curve fitting of vibration response of the concrete (75 µm crumb rubber).

Finally, the equation obtained from the vibration graph (Figure 19) is compared with the reduced general mathematical equation representing the amplitude (Equation (4)), and the damping ratio is obtained as follows:

(6) $$A_{t}=A_{0}\cdot e^{-\zeta \cdot w_{n}\cdot t}$$

where *A_t_* is the amplitude, *A*_0_ is the peak amplitude, *ζ* is the damping ratio, *w_n_* is the natural frequency (rad/s) equal to 2 *πf_n_* where *f_n_* is the natural frequency in Hz, and t is the time in seconds. The calculation of the damping ratio of the SF5%–CR10% sample presented by the graph of Figure 19 has led to 0.0324, which is 3.24% of critical damping ratio.

The second method is used to verify the value obtained from the ECFM and in order to make sure that the tests’ results are more reliable. It is called the logarithmic decrement method (LDM), and the damping ratio in this case is calculated using the logarithmic decrement formulas (Equations (5) and (6)):

(7) $$\delta =\frac{1}{n}ln\frac{A_{0}}{A_{n}}\;if\;n=1,2,3\ldots$$

(8) $$\zeta =\frac{1}{\sqrt{1+{\left(\frac{2\pi }{\delta }\right)}^{2}}}\approx \frac{\delta }{2\pi }$$

where *δ* is the damping index, *A*_0_ is the peak amplitude, *A_n_* is the amplitude after *n* number of cycles, and *ζ* is the damping ratio.

**Figure 20:**
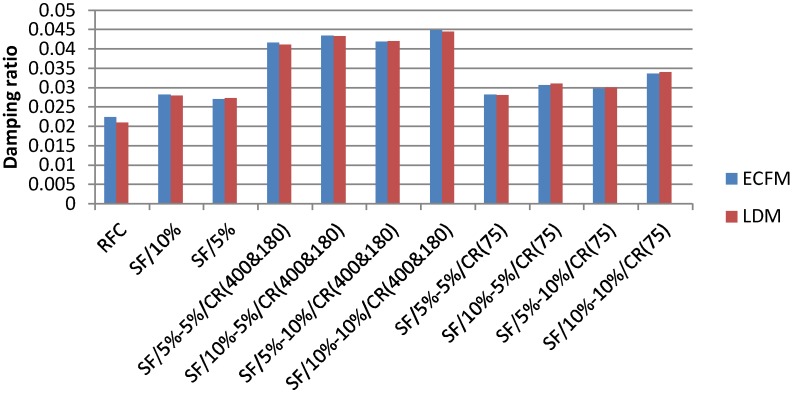
Dynamic damping of crumb rubber concrete at 28 days.

The damping ratio estimated from the LDM for the same sample tested before using the ECFM is 0.0323 (3.23%), which presents a small error of 0.31%. The results regarding all the samples are plotted in the graph of Figure 20. The graph shows that the two methods have similar trend. The reference concrete has the lowest damping ratio with 0.02239 and 0.02098 respectively according to the ECFM and LDM methods. The addition of 10% silica fume improved slightly the concrete’s damping ratio to reach 0.02822 using ECFM and 0.2791 using LDM, and a little more improvement of 19 and 21% respectively when 10% of 75 micron rubber has been added. This improvement is due to the large interface area between the cement matrix and silica fume particles which offer better vibration energy dissipation [57,58]. Finally, the 180&400 micron rubber have proved to have the best effect on the damping ratio of concrete, by reaching the maximum values obtained in this test: 0.04499 using ECFM and 0.04451 using LDM, which present an improvement of 100 and 112% respectively comparing to the conventional concrete. These results confirm the theory stating that the damping ratio of concrete improves with the increase in the size and the amount of rubber content as declared by Zheng at al. [52]. A damping ratio of 3% could easily reduce up to 20–30% of dynamic actions (bending, shear, and force); however, in order to suppress large-amplitude vibrations the damping coefficient should be around 4–5% [57]. Consequently, since the static and dynamic properties of the rubberised concrete (180&400 micron) meet the design standards, it should highly be recommended for concrete railway sleepers’ manufacturing [53,57].

### 5.5. Dynamic Flexural Moduli

**Figure 21:**
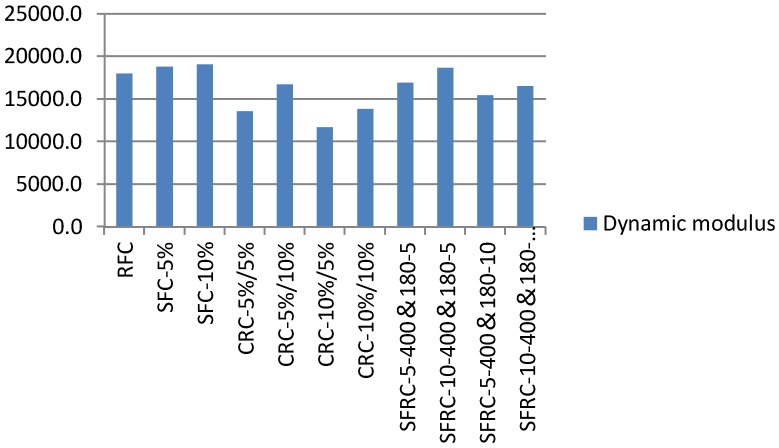
Early-age dynamic moduli of crumb rubber concrete at 7 days (MPa).

**Figure 22:**
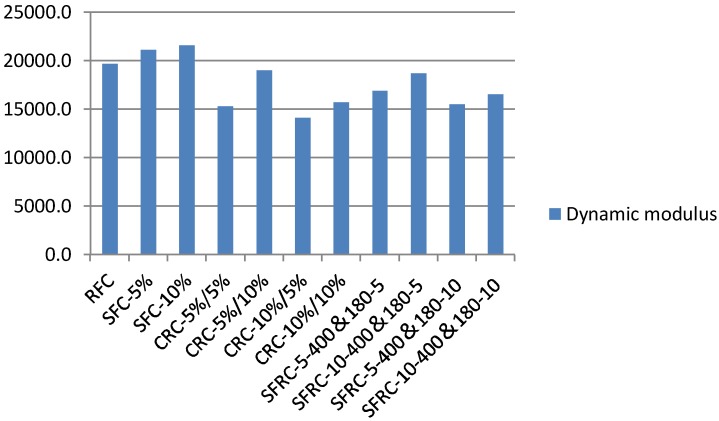
Dynamic moduli of crumb rubber concrete at 28 days (MPa).

According to Kaewunruen et al., the dynamic flexural modulus of elasticity demonstrates the elastic behaviour of material under flexural loading. The dynamic flexural modulus can be estimated from Equation (9) [49,59,60,61]. Figure 21 reveals that the early age dynamic moduli at 7 days of the rubberized concrete are decreased compared to the reference concrete (from 7% to 28%, depending on the mixes). This implies that the release of prestressing force on concrete sleepers should be gentle to minimise the effect on initial deformations (i.e., pre-camber and elastic shortening). Figure 22 shows the dynamic moduli of the concrete at 28 days. It can be observed that the use of silica fume can increase the dynamic moduli at 28 days. The deviation of dynamic moduli of the rubberized concrete is from 5% to 26% compared to the reference concrete. Comparatively, it is found that, at 28 days, the dynamic flexural behavior could be expected to be similar to traditional high-strength concrete (reference). This implies that the difference in creep and shrinkage would be minimal to normal concrete sleepers. These requirements are critical for concrete sleepers in ballasted railway tracks. However, the application of this rubberised concrete can be applied to concrete slabs in ballastless tracks of which its requirement is slightly less stringent [59,60,61,62,63].

(9) $$E_{d}=0.021\rho^{1.5}\sqrt{f_{c}}$$

## 6. Conclusions

Railway traffic environment is aggressive and the railway infrastructure has to experience heavy-haul or high-speed rail operation. Therefore, material for railway infrastructure needs to be strong, durable, resilient and tough to withstand various unexpected damage. Waste tyre is a global environmental problem which is non-biodegradability, flammability and chemical composition. Waste tyre cannot be solved as a normal method like disposal. This research study aimed to develop an environmentally friendly concrete using crumb rubber, recycled form waste rubber tyres (75 micron and 180 micro mixed with 400 micron), as micro-filler to make concrete sleepers that meet with the railway requirements. Although the emphasis is placed on concrete sleepers in ballasted railway tracks, the application of this recycled concrete can also be applied to concrete slabs in ballastless tracks. Based on diverse proportions of crumb rubber and silica fume, eleven different types of concrete were casted according to British standard. The first experiments carried out on the concrete specimens tested their compressive strength at 7 and 28 days, as it presents the most important property of this material. Similar to previous studies’ findings, it has been deducted that the increase in the rubber content results in a drop in compressive strength. However, the rubber concrete containing a proportion of 10% silica fume and 5% crumb rubber has shown considerable results by reaching 57.30 MPa and 59.20 MPa at 28 days in both rubber particle cases 75 micro and the mix of 180 and 400 micro respectively, thus meeting the standard for railway sleepers manufacturing (55 MPa). Likewise, it has been observed a significant deterioration in both splitting tensile and flexural strengths when increasing the crumb rubber proportion in concrete. Still, the proportions that seemed to show better splitting tensile performance than plain concrete are the ones containing 10% silica fume: mixed with 5% crumb rubber in the case of 180 and 400 micro rubber, and 10% in the case of 75 micro rubber (3.75 MPa and 3.54 MPa respectively).

On the other hand, the specimens containing a combination of 10% silica fume and whether 5% of the 180&400 micro rubber or 10% 75 micro rubbers have shown acceptable flexural strengths superior to plain concrete by 8 and 13%: 6.19 MPa and 6.49 MPa, respectively. In terms of electrical resistivity, the concrete samples containing crumb rubber have shown the best results: 374.25 kΩ and 371 kΩ for SF10%/CR10% (180&400) and SF10%/CR5% (75) respectively. Therefore, the addition of crumb rubber is capable of enhancing the electrical resistivity of conventional concrete up to 47%. Finally, by conducting vibration tests on rubberised concrete and using both the exponential curve fitting method (ECFM) and the logarithmic decrement method (LDM), it has been concluded that the crumb rubber has clearly improved the concrete’s damping ratio, especially when using the 180&400 micro which resulted in an improvement of 100% comparing to normal concrete. It is therefore recommended from the results of this study to use crumb rubber in concrete as micro-filler, and more precisely the SF/10%–CR/5% (400&180) as it showed the best performance in all tests and meets the requirements of railway concrete sleepers. From the environmental perspective, the use of crumb rubber in concrete presents one of the best alternatives while dealing with rubber waste, as it will help both the environment protection and also the reduction in railway sleepers cost.

## Figures and Tables

**Table 1:** Chemical and Physical Properties of Undensified Silica Fume [35].

No.	Properties	Value
1	SiO_2_	Minimum 90%
2	Loss of Ignition	Maximum 3%
3	Coarse Particles > 45 μm	Maximum 1.5% (tested on undensified)
4	Bulk Density (U)	200–350 kg/m^3^
5	Bulk Density (D)	500–700 kg/m^3^

**Table 2:** Gradation of aggregates.

No.	Sieves (mm)	Weight Retained (g)	% Retained	Cumulative Retained	% Finer
1	20	0	0%	0	100%
2	16	0	0%	0	100%
3	10	835	21%	21%	79%
4	6.7	2710	67.5%	88.5%	11.5%
5	4.75	355	9%	97.5%	2.5%
6	Base	100	2.5%	100%	0%
	Total	4000 g			

**Table 3:** Concrete mix design.

Main Components						Mixes A	Mixes B
Ingredients kg/m^3^	Cement	Water	Gravel	Sand	Silica Fume SF	Crumb Rubber CR (180 & 400 µm)	Crumb Rubber CR (75 µm)
SF-0%, CR-0%	530	233	986	630	-	-	-
SF-5%, CR-0%	503	233	986	630	27	-	-
SF-10%, CR-0%	477	233	986	630	53	-	-
SF-5%, CR-5%	503	233	986	598	27	32	32
SF-10%, CR-5%	477	233	986	598	53	32	32
SF-5%, CR-10%	503	233	986	567	27	63	63
SF-10%, CR-10%	477	233	986	567	53	63	63

**Table 4:** Types of moulds and corresponding tests.

Mould Type	Number of Samples per Mix	Type of Test
Cube 100 mm	6	Compressive strength
Cylinder Φ 100 × L200 mm	3	Splitting Tensile Strength
Prism W100 × H100 × L500 mm	6	Flexural Strength
Prism W45 × H20 × L120 mm	4	Electrical Resistivity, Vibration, and Damping

**Table 5:** Types of moulds and corresponding tests.

No.	Mixes	Natural Frequency (Hz)				S.D.
		Sample 1	Sample 2	Sample 3	Average	
1	RFC	478	451	486	471.67	±18.34
2	SF/10%	469	427	433	443	±22.716
3	SF/5%	457	437	462	452	±13.23
4	SF/5%–5%/CR (400&180)	476	452	463	463.67	±12.01
5	SF/10%–5%/CR (400&180)	456	439	454	449.67	±9.29
6	SF/5%–10%/CR (400&180)	479	493	474	482	±9.85
7	SF/10%–10%/CR (400&180)	441	446	435	440.67	±5.51
8	SF/5%–5%/CR (75)	443	462	431	445	±15.63
9	SF/10%–5%/CR (75)	477	500	459	477	±20.55
10	SF/5%–10%/CR (75)	453	456	472	460	±10.21
11	SF/10%–10%/CR (75)	488	493	512	498	±12.66
